# what would be needed to start a real primary prevention trial in Alzheimer’s Disease

**DOI:** 10.1002/alz.090514

**Published:** 2025-01-09

**Authors:** Catherine J. Mummery

**Affiliations:** ^1^ National Hospital for Neurology and Neurosurgery, University College London, London, London United Kingdom

## Abstract

Anti‐amyloid therapies are ideal candidates for prevention trials. Secondary prevention in those at risk of ADAD (DIAN‐TU) has shown reduction of brain amyloid deposition leads to significant downstream biological change; early secondary prevention using a monoclonal antibody in sporadic AD (AHEAD 3‐45) is ongoing and will provide critical information on whether treating earlier leads to greater clinical benefit. However, this is not preventing disease but delaying onset in those with presymptomatic disease.

True primary prevention can be defined as treating before any evidence of disease, i.e. amyloid or tau pathology. So how do we identify that stage in sporadic AD? In addition, what is the best mechanism to target for primary prevention? Decreasing production of abeta is a prime candidate.

While there is sound logic in treating prior to disease onset to prevent the disease cascade commencing, there are significant ethical and practical challenges in optimising a prevention trial design. To design a true primary prevention trial in AD, one needs to identify reliably individuals at high risk of developing AD prior to pathology developing. A clear risk triage strategy would be required, alongside development of predictive biomarkers for disease. We need to understand early biological triggers/progression better to be able to define an at risk population and likely timescale to onset of disease. We will need to consider how long to treat for – if not a one‐off treatment, does this potentially commit someone to lifelong treatment? Outcome measures need to be designed to determine the success of a preventative rather than treatment strategy. There have been significant advances in blood biomarkers, but more sensitive clinical and biological markers of early disease onset are required. These trials will be long in duration and require large numbers of participants to commit years to show a treatment effect.

We are at the beginning of prevention trials in AD. In order to succeed, there must be close collaboration between discovery science, industry, the trials community and regulatory bodies to agree a framework that provides the best chance of optimising trial design and maximizing chances of success.

